# Supplementary material for: Are carbon emissions trading and green financial instruments synergistic? -Comprehensive quantitative research based on content analysis
Source: PLoS One. 2024 Mar 7;19(3):e0298601. doi: 10.1371/journal.pone.0298601 (PMC10919601; doi:10.1371/journal.pone.0298601)
Supplement: S1 Data — (ZIP) [file pone.0298601.s001.zip › Data/地级市Z_F工作报告.zip!/║╙─╧/╫ñ┬φ╡Ω╩╨/└┤╫╘╣1⁄2╓┌║┼í╛┬φ┐╦╩2╛▌═°í┐_2009.docx]

各位代表:

现在，我代表市人民政府，向大会作工作报告，请予审议，并请各位政协委员提出意见。

一、 2009年政府工作回顾

刚刚过去的2009年，面对金融危机的影响和经济增速下滑的严峻形势，在省委、省政府和市委的坚强领导下，全市上下深入贯彻科学发展观，认真落实国家和省战危机、保增长的一系列政策措施，紧紧围绕扩投资、拓市场、促消费、强基础四个关键环节，齐心协力，克难攻坚，扎实工作，较好地完成了市二届人大四次会议确定的各项任务，在保增长、保民生、保稳定等方面取得了明显成效。初步核算，全市完成生产总值903亿元，增长11.5%;地方财政总收入51.2亿元，增长11.1 %，其中一般预算收入29.3亿元、支出143.6亿元，分别增长14.5%和32.2%;社会消费品零售总额320亿元，增长19.1%;城镇居民人均可支配收入12311元，增长8.9%;农民人均纯收入4250元，增长9%。一年来，我们突出抓了以下几方面工作:

(一)大力推进重点项目建设。坚持把项目建设作为扩内需、保增长的关键措施，认真实施"4567"投资促进计划，全面实行领导分包重点项目责任制、督查通报制，切实加大协调力度，促进了项目的顺利实施。全市107个重点项目完成投资110.7亿元，占年度计划的102.9%。其中46个计划竣工项目完成投资20.3亿元，占年度计划的101.9%;16个续建项目完成投资58.9亿元，占年度计划的120.3%，石武客运专线、焦桐高速、化新高速等项目进展顺利，薄山水库、宋家场水库除险加固等项目超额完成年度投资计划;35个计划新开工项目中有32个项目开工建设，完成投资30. 2亿元，占年度计划的78.2%;10个前期项目准备工作顺利推进。新增中央投资项目按计划顺利实施。全年完成城镇固定资产投资408.7亿元，增长32.2%，投资对经济增长的贡献份额达65%。

(二)着力抓好工业经济发展。针对市场需求萎缩、企业效益下滑的实际，及时筛选872户企业作为市县重点服务对象，深入开展企业服务年活动，逐企业明确分包领导、建立问题台帐，认真研究解决问题，切实加强运行调节，促进了工业经济的平稳发展。全市规模以上工业完成增加值225.7亿元,增长17.6%;主营业务收入838亿元，增长18.8%;实现利润55.2亿元，增长13.5%;实现税金32.9亿元，增长20.6%;工业用电量达40.8亿千瓦时，增长8.7%。扎实推进自主创新工作，4家企业申报省级工程技术研究中心，7家企业被认定为省级科技创新型企业。认真落实节能减排措施，万元GDP能耗下降4.63%，二氧化硫、化学需氧量排放分别削减0.2万吨和1万吨，全面完成了省定目标任务。加快推进产业集聚区建设，完善基础设施，创新管理体制，增强了吸纳集聚能力，全市12个产业集聚区全部完成总体规划编制、上报工作，总规划面积达150.6平方公里，累计完成投资97.1亿元，入驻企业740家。

(三)坚持不懈地做好"三农"工作。全面落实各项强农惠农政策，继续加大"三农"投入，保持了农业农村发展的好态势。全年共发放粮食直补、综合补贴资金9.4亿元，兑现良种补贴和农机具购置补贴资金2.44亿元。积极实施抗旱水源、防洪除涝、灌区配套等项目建设，新增和恢复有效灌溉面积132万亩，新增旱保田33万亩。粮食总产达到66.17亿公斤，增长1.7%，再创历史新高。畜牧业总产值172.6亿元，增长6 %，肉、蛋、奶产量分别达到76.6万吨、32.1万吨和5.99万吨。新建、完善通道绿化2044公里，成片造林61万亩。各类农业产业化组织发展到1240家，省、市重点龙头企业分别达到21家、206家。完成了102个贫困村整村推进和12.7万人的脱贫任务。新发展农村沼气5万户，解决了53万农村人口的饮水安全问题。培训农村劳动力10万余人，实现劳务输出221万人，劳务收入达120亿元。

(四)努力加快城镇化步伐。城市空间发展战略规划和"三区两县一体化"规划编制工作顺利开展，中心城区控制性详规编制工作进度加快。驿城大道、练江路西段、中华路西段、铜山大道北段和练江河、冷水河治理等工程顺利实施，新建、改造了一批游园、广场和环卫设施。实施了36个旧城改造和城中村改造项目。中心城区完成投资26亿元，建成区面积达到50多平方公里。县城和小城镇供水、供电、通信、道路等基础设施逐步改善，吸纳集聚能力明显增强。全市城镇化率达到29.5%,比上年提高1.8个百分点。深入开展"四城联创"活动，切实加强城市管理，抓好绿化、美化、净化，成功创建了中国优秀旅游城市。大力发展第三产业，全年完成投资226.2亿元，增长40.5%，其中房地产开发投资70.7亿元，增长55.9%。金融机构各项存款余额794.39亿元、贷款余额419.82亿元，分别增长20.7%和25.2%，被评为中国最具发展潜力金融生态示范城市。

(五)继续深化改革开放。国企改革顺利推进，水管体制改革圆满完成，文化事业单位改革全面启动，集体林权改革取得阶段性成效。交通系统改革稳步实施，交通规费征稽和公路通行费收费人员实现了有序转岗。坚持以东西合作为载体，借鉴宿迁等地经验，强化了招商措施，完善了奖惩机制，先后组织了赴福建泉州、广东东莞等地招商活动，有力推动了招商引资的顺利开展。成功举办了全国农产品加工业博览暨东西合作投资贸易洽谈会，签约500万元以上合作项目556个，合同资金361亿元。全年累计引进新项目443个，合同金额228亿元，落实到位资金73.5亿元。全市新批外商投资企业19家，实际利用外资8366万美元，增长15.6%;完成出口12911万美元，增长9.3%。

(六)扎实推进和谐社会建设。市里确定的"十五大实事"得到较好落实。全年新增城镇就业8万人，下岗人员再就业2.3万人，其中"4050"人员9216人，城镇登记失业率控制在3.6%。社会保障标准不断提高，覆盖面持续扩大，城乡低保人数达到46.3万人，城镇居民医保参保人数达到68.8万人，新农合参合率达94.6%，平舆农村新型社会养老保险试点顺利启动。开工建设廉租房、经济适用房36.8万平方米和84.5万平方米，发放廉租房租赁补贴资金2393万元。实施各类科技计划项目163项，研发新技术、新工艺180项。落实农村义务教育"两免一补"资金7.44亿元、城市中小学免除杂费资金1322.8万元。中小学布局调整稳步推进，新建和扩建了一批中小学校。积极实施职业教育攻坚计划，职教园区建设顺利启动。23个乡镇综合文化站、210个农家书屋、796个基层网点建设全面完成。市广电中心、工人文化活动中心、博物馆、群艺馆等工程建设顺利推进。改造了一批乡镇卫生院、城市社区卫生服务中心，新建标准化村卫生室56所、改扩建870所。积极开展白内障无障碍市创建工作，完成白内障手术2.51万例。扎实做好艾滋病防治救助工作，有效控制了手足口病、甲型H1N1流感疫情。认真做好计划生育工作，人口自然增长率控制在5.32‰。交通事业快速发展，高速公路、农村公路分别完成投资16.36亿元和7.87亿元。加强食品药品监管，保障了人民群众的健康安全。扎实推进平安建设，认真落实安全生产责任制，狠抓事故隐患排查治理，有效防止了重大安全事故的发生，事故起数和死亡人数分别下降29.7%和27%。切实加强社会治安综合治理，严厉打击各种刑事犯罪行为，进一步规范信访秩序，积极化解各类矛盾，妥善处置群体性事件，保持了社会大局的和谐稳定。人事、审计、旅游、统计、体育、气象、民族宗教、外事侨务、红十字会、史志、档案等工作都取得了新的成绩。援助四川江油灾后重建项目一期工程顺利竣工。

(七)切实加强民主法制和精神文明建设。深入开展学习实践科学发展观和"讲、树、促"等活动，扎实推进"两转两提"，服务型政府建设迈出新的步伐。坚持依法行政，自觉接受人大及其常委会监督，支持政协参政议政，认真办理人大代表建议及政协提案。加强政府系统廉政建设，搞好行政监察，创新政风行风评议模式，政风行风明显好转。坚持以创建省级文明城市为载体，深入开展各类群众性精神文明创建活动，公民道德水平和城乡文明程度继续提升，有望进入创建省级文明城市先进市行列。高度重视驻驻部队、国防后备力量和人民防空建设，认真做好拥军优属、拥政爱民和军民共建工作，军政军民团结进一步巩固，再次荣获全省"双拥模范城"称号。

各位代表!过去的一年，在巨大的困难和挑战面前，我市经济社会发展取得这样的成绩极其不易。这是省委、省政府和市委科学决策、正确领导的结果，是市人大、市政协和社会各界积极支持的结果，也是全市人民团结奋斗的结果。在此，我代表市人民政府，向工作在全市各条战线的广大工人、农民、知识分子、干部、驻驻部队指战员、武警官兵、公安民警以及各界人士致以崇高的敬意!向所有关心、支持驻马店建设和发展的同志们、朋友们表示衷心的感谢!

在肯定成绩的同时，我们也清醒地看到，当前我市经济社会发展仍然面临不少困难和问题。一是经济总量小，人均水平低，社会事业欠账多，财政收支矛盾突出，一些涉及群众切身利益的问题还没有得到很好解决，保障和改善民生的任务仍很艰巨。二是经济结构不合理，产业层次低，发展方式粗放，部分行业和企业生产经营比较困难，经济增长的质量和效益不容乐观。三是城镇化发展滞后，工业化水平低，农村基础设施薄弱，以城带乡、以工补农能力不足，统筹城乡发展的任务十分繁重。四是有些干部思想观念陈旧，不能适应形势的需要，少数部门发展意识不强，服务意识差，办事效率低，影响了行政效能的发挥。对此，我们一定高度重视，采取有效措施认真加以解决，决不辜负全市人民的期望和重托。

二、2010年政府工作总体要求和奋斗目标

今年是实施"十一五"规划的最后一年。做好今年经济社会发展工作，对夺取应对危机全面胜利、保持经济平稳较快发展、为"十二五"规划启动实施打好基础意义重大。总体上看，今年经济发展环境将好于去年。世界经济正在复苏，国内经济回升向好，国家继续实行扩大内需政策，进一步加大对"三农"的投入，促进中部地区崛起规划全面实施，产业转移趋势更加明显。特别是经过近些年的发展，我市的资源、区位和市场优势正在扩大，基础设施不断完善，综合实力显著提升，广大干部群众干事创业的积极性逐步高涨，形成了较好的发展趋势、态势和气势。同时，我市经济社会发展面临的形势依然十分复杂。国际金融危机的影响还会持续，国内经济回升的动力依然不足，不确定、不可预料因素也在增多，刺激消费和投资增长的政策效应可能减弱，加之我市自身存在的突出矛盾和困难，都严重制约着平安崛起的步伐，给我们的工作带来前所未有的压力。因此，我们既要看到发展中的诸多有利条件，增强机遇意识，进一步坚定必胜信心;又要时刻保持清醒头脑，增强忧患意识，更加周密地积极应对困难和挑战，努力把我市经济社会发展推上一个新台阶。

今年政府工作的总体要求是:认真贯彻党的十七届三中、四中全会精神，深入贯彻落实科学发展观，认真落实省委和市委经济工作会议各项部署，按照"重在持续、重在提升、重在统筹、重在为民"的要求，继续深化"三项建设"，加快工业化、城镇化和农业现代化进程，提升产业层次，促进经济转型，着力提高经济发展的质量和效益，努力实现项目建设、工业发展有新提升，农村经济、城镇建设有新进展，改革开放、招商引资有新突破，改善民生、促进和谐有新成效，推动全市经济社会又好又快发展。

今年经济社会发展的主要目标是:生产总值增长12%，规模以上工业增加值增长15%以上，城镇固定资产投资增长30%，社会消费品零售总额增长16%，地方财政总收入增长8 %，其中一般预算收入增长12%，城镇登记失业率控制在4.4%以内，城镇居民人均可支配收入增长8%，农民人均纯收入增长6%，人口自然增长率控制在6‰以下，单位生产总值能耗、化学需氧量和二氧化硫排放量控制在省下达目标以内。

围绕上述总体要求和主要目标，全面完成今年的各项目标任务，我们必须始终把发展作为第一要务，坚持"三个结合"，实施"三百工程"，实现"三个突破"，落实"三个确保"。坚持"三个结合":一是坚持把保增长与改善民生、调整结构结合起来。把保持经济平稳较快增长作为首要任务，注重在发展中调整，在调整中发展。同时，通过发展经济，切实保障和改善民生，通过改善民生，充分调动人民群众发展的积极性。二是坚持把项目建设与扩大消费、增加出口结合起来。在继续抓好项目建设的同时，努力挖掘消费潜力，扩大出口份额，不断提升投资和消费对经济增长的拉动作用。三是坚持把扩大开放与深化改革、创新体制机制结合起来。进一步解放思想，大胆探索，积极推进各项改革，努力提高开放的层次和水平，不断增强经济社会发展的动力和活力。实施"三百工程":在工业、农业、基础设施、城市建设、服务业等领域实施300个总投资3000万元以上的结构调整和基础设施项目，投资总规模超过600亿元。实现"三个突破":地区生产总值突破1000亿元，城镇固定资产投资总额突破500亿元，二三产业增加值占GDP的比重突破75%。落实"三个确保":确保经济发展目标圆满完成，确保民生工程顺利实施，确保社会大局和谐稳定。

三、2010年政府工作主要任务

做好今年的政府工作，必须立足当前，着眼长远，统筹兼顾，突出重点，强化措施，狠抓落实，努力推进经济社会协调发展。

(一)着力保持投资较快增长

坚持以项目建设为总抓手，通过抓好在建项目、开展招商引资、积极向上争取项目、启动民间投资等，继续扩大投资规模，进一步增强投资对经济增长的拉动作用。今年初步安排重点建设项目105个，总投资532.9亿元，年度计划投资116.2亿元。工业方面，加快国电2×30万千瓦供热机组、豫龙同力日产4500吨水泥熟料、昊华骏化年产30万吨纯碱和30万吨氯化氨、华顺阳光太阳能、徐福记食品加工、维维粮油等计划竣工项目建设进度，确保按期竣工投产;抓好顺达化工年产36万吨醋酸改扩建、昊华骏化3万吨三聚氰胺、白云纸业高档文化纸、大程面粉等续建项目建设，确保完成年度建设任务;开工建设华润安里煤矿、昊华骏化30万吨合成氨、十三香工业园等项目。做好华润电力风力发电、天瑞水泥等项目的前期工作。水利方面，加快宿鸭湖除险加固、杨庄滞洪区安全、灌区续建配套节水改造项目建设进度，完成谭山等14座中小型水库除险加固任务，开工建设蛟停湖滞洪区安全、重点平原洼地治理等重大工程项目。交通方面，抓好京港澳高速驻马店段改扩建、焦桐高速泌阳段、化新高速及石武客运专线等重点项目建设，继续实施干线公路大中修和农村公路建设。积极推进境内军民合用机场建设。社会事业方面，全面完成市广电中心、工人文化活动中心、博物馆、群艺馆等文化设施建设，加快市中心医院病房大楼、市第二中医院迁建工程等项目建设步伐，开工建设市体育馆、市体育中心、市中心医院新区分院和市精神病院病房大楼。进一步落实项目协调推进机制，完善联审联批制度，继续开展项目观摩点评活动，激发各地抓项目建设的积极性、主动性。大力优化项目建设环境，在土地、资金、环境容量等方面优先保障重点项目建设。搞好项目筛选，严格控制低水平重复建设和高污染、高耗能项目上马。继续加强银企合作，支持企业战略重组和上市融资，认真落实国债项目配套资金，确保项目建设的资金需求;加强对财政资金投入项目的及时跟进、全程监督，防止出现"半拉子"工程和豆腐渣工程。抓好项目储备库建设，结合"十二五"规划编制，围绕国家投资重点，认真做好项目筛选论证工作，高起点谋划一批大项目、好项目，加快前期工作进度，争取更多的项目进入国家、省发展规划。

(二)大力推进发展方式转变

在促进工业发展的同时，更加注重提升层次，优化结构，加快转型，着力提高发展的质量和效益。加快工业结构优化升级。继续支持重点企业开展合资合作，壮大企业规模，提升产业层次，促进优势企业做大做强。全面实施质量兴市战略，引导企业加大技术改造力度，鼓励企业应用新技术、新工艺、新材料、新设备，大力培育名优企业和名牌产品。积极引导资金、技术、人才等要素资源向优势产业集中，加快能源和煤化工、食品、建材、医药、装备制造等支柱产业发展，不断壮大产业集群，带动全市产业布局优化、结构升级。加强自主创新能力建设。大力培育创新型企业和高新技术企业，积极筹建科技研发公共服务平台，搞好科研攻关，努力开发一批具有自主知识产权的新产品，提高企业的核心竞争力。支持天方药业争创国家级工程技术研究中心，白云纸业建立省级研究中心，力争建立8个以上市级研究中心。加快产业集聚区发展。建立多元化的投入机制，加快完善园区水、电、路、通讯等配套设施和技能培训、信息咨询、文化教育等公共服务体系，创新管理体制机制，落实好财政激励、优惠电价、税收分成、建设用地等优惠政策，继续提高集聚区承载力、吸引力和竞争力。引导老城区企业"退二进三、入园进区"，促进产业集群发展。继续强化对企业的服务。逐步建立市场开拓、要素保障、环境优化、困难企业帮扶等服务企业的长效机制，推动服务企业工作常态化。市、县两级继续筛选一批重点企业作为服务对象，健全服务制度，完善服务措施，提高服务效率和水平。加强企业运行调控调节，搞好煤、电、油、运、气的供需调度，努力提高保障能力，确保企业生产稳定有序。强化节能减排和生态环境建设。坚决落实节能减排目标责任制和"一票否决制"，进一步强化工作措施，严格执行产业政策，突出抓好高耗能行业和重点领域的节能降耗，加强重点流域、重点区域、重点行业污染综合整治，抓好污水、垃圾处理设施建设和运营，确保完成节能减排目标任务。继续推进林业生态建设，确保完成造林29.51万亩，完善农田林网 100万亩，力争完成林业生态县、生态市创建任务。实行最严格的耕地保护制度，大力推进土地综合整治，严格落实城乡建设用地增减挂钩政策，切实提高土地综合利用效率。

(三)继续加强"三农"工作

坚持以粮食核心区建设为契机，全面落实强农惠农政策，加快发展现代农业，扎实推进新农村建设，促进农业发展、农民增收和农村和谐。巩固提高粮食综合生产能力。落实好农民种粮直接补贴和主要粮食品种最低收购价政策，保护和调动农民种粮积极性。扎实推进粮食核心区建设，重点抓好一批大中型灌区续建配套、中低产田改造、农田排灌工程和高标准农田示范工程建设;加快6个农业综合开发重点县、9个产粮大县以及8个县区土地治理等项目建设，着力稳定和扩大粮食种植面积，确保粮食总产稳定在125亿斤左右。加大农业科技创新和推广力度，加快农业机械化进程，充分挖掘农业增产潜力。深入推进农业结构调整。紧紧围绕食用菌、蔬菜、花木等产品，因地制宜发展特色农业，打造农业品牌，培育产业优势。继续优化畜牧业结构，加强重大动物疫病防控，抓好生猪调出大县、肉牛发展大县和标准化规模养殖场(小区)建设，促进现代畜牧业发展。大力扶持农产品精深加工业，积极培育壮大龙头企业，推动农业产业化发展。加快农产品质检项目和产业技术体系建设，提高农业标准化水平。积极发展农民专业合作组织和经纪人队伍，不断提高农业生产组织化程度。加大农村劳动力培训力度，完成10万人次的培训任务。加强劳务输出的组织、引导和管理，鼓励外出务工成功人士回乡创业，带动农民致富。继续改善农村生产生活条件。增加并整合支农资金，继续推进农村基础设施建设，力争解决40万农村人口的安全饮水问题，新增农村沼气用户2万户，完成10万农村贫困人口的脱贫任务;新增和恢复有效灌溉面积40万亩，新增旱保田30万亩，新增和改善除涝面积12万亩。加快村镇规划编制工作进度，以20个省级和50个市级新农村建设示范村为重点，扎实推进新型农村住宅社区建设。积极推进农村改革。加强土地承包经营权流转管理和服务，在依法自愿有偿基础上，鼓励发展多种形式的适度规模经营。深化农村信用社改革，加快组建农村商业银行。加快县级供销社改革。全面推进农村集体林权制度改革，基本完成集体林权确权发证任务。

(四)努力提高城镇综合承载能力

牢固树立统筹城乡发展的理念，多渠道融通资金，加强城镇建设和管理，增强城镇吸纳和辐射带动能力，促进城乡互动、良性发展。坚持大手笔规划。统筹产业、城镇、交通、生态等各类空间要素，加快编制中心城区空间发展战略规划，结合新一轮土地规划修编，完成"三区两县一体化"总体规划和各专项规划的编制及评审工作。继续完善中心城区道路、水系、市场、学校、医院、车站等公共服务设施规划。严格城镇规划管理，严禁乱搭乱建，坚决清理和拆除违章建筑，切实维护规划的严肃性。坚持高标准建设。抓好驿城大道北段、铜山大道中段、解放大道西段及汝河大道、中原大道等市政道路建设和改造，加快练江河、冷水河等水系治理进度，启动老火车站广场、石武高铁火车站广场、公交北停车场改造建设。加大旧城和城中村改造力度，抓好环卫设施、园林绿化工程建设和管理，着力改善人居环境，努力实现国家园林城市创建目标。加快县城和重点镇建设，完善城镇功能，强化产业支撑，逐步把县城发展成为常住人口20万人以上的中等城市。坚持高水平管理。深入开展"四城联创"活动，建立健全城市管理长效机制，实施精细化、规范化管理，确保市容整洁、环境优美、秩序井然。继续加大城镇环境综合整治力度，切实解决城中村、城乡结合部、国道沿线村镇"脏乱差"现象，继续改善城乡环境面貌。创新城市发展体制机制。全面推进城镇建设投融资体制改革，在扩大政府投资的同时，进一步开发城镇基础设施建设和产业发展项目，努力争取国债资金和金融机构的信贷支持。积极运用市场机制，鼓励和引导金融机构、民间资本多方参与城市建设。不断拓展特许经营、转让经营权等方式，吸引更多的外商投资城建项目。加快城镇户籍管理、社会保障制度改革，放宽城镇落户条件，引导在城镇稳定就业和居住的农民有序转变为城镇居民。

(五)深入推进改革开放

牢固树立大开放、大发展意识，敢于破除束缚发展的体制障碍和陈旧观念，善于在改革创新中寻找出路、谋求发展。继续深化改革。积极稳妥推进政府机构改革。继续推进国企战略重组及地方铁路、商贸系统等非工业企业改革，加快市直局属企业脱钩改制步伐。毫不动摇地鼓励、支持、引导非公有制经济发展。支持市商业银行跨区经营，确保成功创建三级银行。完善基本医疗保障制度，全面实现医疗救助与新农合、城镇职工和居民医保同步结算。扩大国家基本药物制度覆盖面，构建药品供应保障体系。推进公共卫生、基层医疗卫生事业单位实行绩效工资。全面完成市影剧院、豫剧团、电影放映公司转企改制和市、县文化市场综合执法改革任务。深入开展大招商活动。依托产业集聚区，建设一批承接产业转移的示范园区，吸引沿海地区食品、纺织服装、中小型电子等劳动密集型产业向我市转移。紧紧围绕支柱产业、优势骨干企业的发展，加强对外交流与合作，组织开展好一系列重大招商引资活动，努力提高招商质量和利用外资水平。继续做好今年东西合作会的筹办工作，创新办会形式，提升办会层次，力求取得更大的成效。加强对已签约项目的跟踪落实，切实提高项目履约率、资金到位率和开工投产率。强化招商引资责任，严格考核，兑现奖惩，确保全市招商引资工作实现新突破。积极扩大出口。落实稳定和鼓励出口的政策措施，重点支持优势农畜产品、医药、化工、帽服等传统产品的出口，逐步提高机电产品、高新技术产品的出口规模。加快口岸建设，建立快速商检、通关机制，降低通关成本，促进外贸发展。

(六)积极扩大消费需求

坚持把扩大消费和改善民生相结合，努力提高消费对经济增长的拉动作用。认真落实刺激消费政策。全面落实国家调整国民收入分配的政策，加强企业工资集体协商和工资支付保障制度建设，落实最低工资制度，提高企业退休人员基本养老金、部分优抚对象待遇和城乡居民最低生活保障水平。继续落实"汽车摩托车下乡"、"家电下乡"等优惠政策,促进农村市场消费。加强和改善房地产市场调控，支持中低价位商品房建设，加大保障性安居工程建设力度，力争完成30万平方米经济适用房、37万平方米廉租房建设任务。积极培育新的消费热点。继续实施万村千乡、双百市场工程和农超对接，建设、改造一批农村便民超市、配送中心和农产品批发交易市场。大力推动文化、健身、培训、家政、养老等服务性消费，做大做强假日消费市场，促进消费结构优化升级。深入整顿和规范市场秩序，强化食品、药品监管，严厉打击制假售假、强买强卖、哄抬物价等违法行为，为扩大消费创造良好环境。大力发展服务业。积极改造提升商贸、餐饮、交通运输等传统服务业，大力发展现代物流、休闲娱乐、市场中介等新兴服务业。加强物流基础设施建设，积极发展综合物流和行业物流，加快推进中原农业产业经济城建设，力争尽快建成投用。积极培育文化企业、文化市场，推进文化产业发展。以成功创建中国优秀旅游城市为契机，加快嵖岈山、铜山、薄山湖、南海寺、竹沟红色旅游等景区建设，加强宣传推介，推进资源整合，完善基础设施，提升景区品位，努力打造旅游精品线路，使旅游业成为新的经济增长点。切实加强财政金融工作。加强税收征管和非税收入管理，优化支出结构，严格控制和压缩一般性支出，确保有限的财力用到最需要的地方。认真落实国家货币信贷政策，加强对重点领域和薄弱环节的资金支持。加快村镇银行、小额贷款公司、中小企业担保机构建设，提高市县政府投融资平台融资能力，着力缓解中小企业融资难问题。

(七)继续加强和谐社会建设

坚持把改善民生摆在更加突出的位置，集中力量办好"十五大实事"，切实让发展成果惠及广大人民群众。加强就业和社会保障工作。坚持把就业作为民生之首，继续实行积极的就业政策，将就业扶持优惠政策再延长一年。积极开发公益性就业岗位，帮助就业困难人员就业，动态消除"零就业家庭"问题，确保全年新增就业人员4.2万人，实现下岗失业人员再就业1.1万人，解决"4050"人员就业4000人。深入开展全民创业活动，加强创业培训和辅导，力争实现以创业带动就业4.4万人。积极做好高校毕业生和复转军人就业安置工作。继续扩大企业养老、医疗、失业、工伤和生育保险覆盖面，落实养老保险省级统筹转移接续政策，基本解决各类关闭破产企业退休人员和困难企业职工医疗保障问题，基本实现工伤保险市级统筹。抓好新型农村社会养老保险试点，完善被征地农民补偿机制和社会保障制度。认真落实困难群众救济救助制度，切实抓好城乡低保、农村五保集中供养和残疾人康复进农村进社区、职业培训及危房改造等工作。积极发展社会福利和慈善事业。加强保障性住房建设，加大廉租住房租赁补贴发放力度，着力解决低收入居民住房困难。大力发展各项社会事业。加大农村教师培训选聘力度，实施好中小学校舍安全、农村初中校舍改造等项目，完成1500所农村公办完全小学布局调整任务，加强中心城区、县城中小学规划和建设，实施城镇支教工程，推动义务教育均衡发展。大力推进素质教育，减轻中小学生课业负担。继续解决好进城务工人员随迁子女平等接受义务教育和留守儿童义务教育问题。强力推进职业教育攻坚，加快职教园区和实训基地建设，基本完成职教资源整合工作。加强医疗卫生基础设施建设，支持一批县级医院、乡镇卫生院、计生服务中心、社区卫生服务中心和村卫生室建设改造。继续抓好艾滋病防治救助、甲型H1N1流感防控等工作。切实抓好乡镇综合文化站、农家书屋、广播电视村村通、无线数字电视家家通等项目的实施。深入开展全民健身运动，积极备战第七届全国农民运动会和省第十一届运动会。继续实施农村部分计划生育家庭奖励扶助和特别扶助制度，综合治理出生人口性别比偏高问题，稳定低生育水平。继续做好人事、审计、统计、外事、档案、侨务、史志、地震、气象等工作。切实加强精神文明建设。加强社会主义核心价值体系建设，大力弘扬以爱国主义为核心的民族精神和以改革创新为核心的时代精神。以开展"三优三创"竞赛活动和"清洁家园行动"为载体，推动文明城市、文明村镇、文明单位等群众性精神文明创建活动的蓬勃开展。加强诚信体系建设和社会公德、职业道德、家庭美德教育。加强法制宣传教育，积极开展法律援助。继续推进文化科技卫生"三下乡"、"四进社区" 和舞台艺术送农民等活动，进一步提高城乡居民的文明程度。全力维护社会和谐稳定。深入开展平安建设，认真落实安全生产责任制，着力抓好重点行业和重点领域的安全监管，防止重特大事故发生。深入推进社会管理创新，正确处理各类社会矛盾，坚决纠正损害群众利益的突出问题，保障群众合法权益。加强和改进信访工作，进一步完善应急机制，积极预防和妥善处置群体性事件和突发公共事件。继续加强社会治安综合治理，健全社会防控体系，依法防范和严厉打击各类违法犯罪活动，进一步增强人民群众的安全感。全面贯彻党的民族宗教政策，切实做好民族宗教工作。加强社区建设，完善村民自治。加强国防教育和国防后备力量建设，支持驻驻解放军和武警部队建设，不断提高预备役部队、民兵和人民防空建设质量;积极开展拥军优属和双拥活动，巩固军政军民团结。全面完成援助四川江油灾后重建任务。

(八)切实加强政府自身建设

深入开展学习实践科学发展观活动，扎实推进"两转两提"，坚持科学性，增强紧迫性，突出实效性，努力建设为民、责任、务实、高效、廉洁政府。坚持依法行政。严格依照法定权限和程序履行职责，努力为各类市场主体创造公平发展环境，为人民群众提供良好公共服务，维护社会公平正义。严格执行人大及其常委会的决议、决定，自觉接受人大法律监督和政协民主监督，认真办理人大代表建议和政协提案，充分听取各民主党派、工商联、无党派人士及各人民团体的意见，强化监察、审计等专门监督，广泛接受社会公众和新闻舆论的监督。讲求实干实效。牢固树立宗旨意识，坚持求真务实、执政为民，坚决克服官僚主义、形式主义，反对急功近利、虚报浮夸。尊重规律，科学决策，努力做到不走错路、少走弯路。完善考核评价机制，少提口号、多干实事，说到做到、说好做好。大力整治文风会风，严格控制各类检查评比和节庆活动，集中精力谋大事、谋发展，抓实事、抓落实，努力创造出经得起历史和人民检验的业绩。提高服务效能。深化行政管理体制改革，进一步减少和规范行政审批。加强和规范行政服务中心建设，认真落实联审联批、代办制、首问责任制、限时办结制和责任追究制，逐步做到一个机构对外、一个机构履职。强化政风行风评议，加强对市县政府部门内设机构负责人的监督考评，强化行政问责，重点整治行政不作为、乱作为等行为，努力提高服务质量和行政效率。加强廉政建设。坚持标本兼治、综合治理、惩防并举、注重预防的方针，严格落实廉政建设责任制。健全公共资源配置、公共资产交易、公共产品生产等领域的管理制度，推进权力运行程序化，从源头上防治腐败。依法推进政务公开，主动接受各方面监督，让权力在阳光下运行。探索建立系统规范的财政预算公开机制，逐步将公务消费纳入政务公开范围。坚决查处违法违纪案件，切实纠正损害群众利益的不正之风，以反腐倡廉的实际成效取信于民。

各位代表!面对改革发展的新形势，面对全市人民的新期待，我们深感责任重大、使命光荣。让我们在省委、省政府和市委的坚强领导下，深入贯彻落实科学发展观，坚定信心，迎难而上，扎实工作，凝聚全市人民的智慧和力量，为实现驻马店平安崛起而努力奋斗!
